# Digital opportunities to connect and complain – the use of Facebook in small animal practice

**DOI:** 10.1002/vro2.29

**Published:** 2022-02-28

**Authors:** Svenja Springer, Thomas Bøker Lund, Peter Sandøe, Sandra A. Corr, Annemarie T. Kristensen, Herwig Grimm

**Affiliations:** ^1^ Unit of Ethics and Human‐Animal Studies, Messerli Research Institute, University of Veterinary Medicine Vienna, Medical University of Vienna University of Vienna Vienna Austria; ^2^ Department of Food and Resource Economics University of Copenhagen Frederiksberg Denmark; ^3^ Department of Veterinary and Animal Science University of Copenhagen Frederiksberg Denmark; ^4^ Division of Small Animal Clinical Sciences, School of Veterinary Medicine University of Glasgow Glasgow Scotland; ^5^ Department of Veterinary Clinical Sciences University of Copenhagen Frederiksberg Denmark

**Keywords:** complaints, Facebook, questionnaire survey, small animal practice, social media, veterinary medical ethics

## Abstract

**Background:**

Social media is increasingly used in small animal practice, enabling veterinarians to connect with clients and promote their business online. It can also be used by clients to quickly distribute complaints via online communities.

**Material/methods:**

Using a questionnaire study we investigated Austrian, Danish and UK veterinarians’ attitudes towards Facebook, the contents of clients’ online complaints and how they were handled by veterinarians (*N* = 648).

**Results:**

In Denmark and the UK, around 90% of practices had a Facebook page, in contrast to 40% of Austrian practices. Most Danish and UK veterinarians agreed that the use of Facebook was relevant and expected by clients. Agreement was lower among Austrian veterinarians, probably reflecting the lower uptake of social media there. In particular, younger veterinarians and those who actively used Facebook for the practice, could see benefits. In all three countries, we found that clients most frequently complained about treatment costs. Most veterinarians preferred to actively deal with clients’ complaints, either replying online or discussing them directly.

**Conclusions:**

We recommend future research focusing on veterinarians’ personal use of social media and on clients’ use of and attitudes towards social media in the veterinary context.

## INTRODUCTION

Social media platforms are considered by many to be an important way to connect with other people. Platforms such as Facebook allow professional people and/or institutions to advertise their services, share information, engage in dialogue and answer questions.^1^, ^2^, ^3^ Following this trend, veterinarians increasingly make use of social media to raise their profile and promote their business online.^1^, ^2^, ^3^, ^4^, ^5^, ^6^, ^7^

Compared to other means, social media platforms stand out in terms of affordability and the opportunity to reach specific groups of interested people.^8^ That in turn promises to increase engagement by promoting trustful and long‐standing relationships between the client and the veterinarian and/or the veterinary practice.^8^ Conversely however, dissatisfied clients can use social media platforms to complain about the veterinary practice. This creates challenges including the time required to respond to negative feedback and the potential negative consequences on the reputation of the practice.^2^, ^9^, ^10^ The management of negative feedback on social media can be influenced by at least two important factors: first, the relevant experience of the person dealing with the complaint and second, the number of people who have access to the social media platform.

Previous studies with animal owners investigated the use of social media to gather information about pet health.^7^, ^11^, ^12^ For example, in a study from the US, UK and Canada, approximately half of dog and cat owners used Facebook to obtain pet health information.^12^ Furthermore, over 50% of dog and cat owners used Facebook to post pet health information for other Facebook users.^12^ Whereas previous studies mainly focused on the animal owner's use of social media and attitudes towards it,^11^, ^12^ to the best of the authors’ knowledge, no studies have examined veterinarians’ attitudes towards and use of social media in small animal practice. Previous publications on this topic have been based only on anecdotal knowledge,^1^, ^2^, ^4^, ^6^, ^10^, ^13^, ^14^ presented results of Facebook pages of veterinary practices,^8^, ^9^ reported on the general usefulness of social media for veterinarians,^3^ or advised on what information should be conveyed.^15^ Further, although studies have indicated that veterinarians are increasingly challenged with online complaints,^2^, ^9^, ^10^ the contents of complaints and how veterinarians actually handle them has also not been investigated.

This questionnaire study involved small animal veterinarians from Austria (AT), Denmark (DK) and the UK, to provide insights into veterinarians’ attitudes towards Facebook, the content of client's online complaints and how veterinarians handle them. The use of Facebook, the largest online social network worldwide,^16^ differs among the three countries. In Austria, 44% of the population use Facebook,^17^ while 66.7% (in April 2020) and 69.8% (in April 2020) use Facebook in the UK and Denmark, respectively.^18^, ^19^ It is likely that these differences not only correlate with the use of Facebook by veterinary practices, they will also impact on veterinarians’ attitudes towards this use. Further, the attitudes of younger veterinarians might differ in comparison to older veterinarians, since members of the former grew up with online networks and are more familiar with social media. Insofar as negative feedback on social media and/or webpages is concerned, it seems likely that veterinarians’ work experience, employment status or the type of practice (e.g. independently owned versus corporate owned) in particular might explain differing ways of handling online complaints.

The main objective of this study was to answer the following research questions: (i) What are veterinarians’ attitudes towards the use of Facebook and how are these attitudes affected by socio‐demographic and practice‐specific factors? (ii) How often do veterinarians find complaints from clients about their services on webpages and/or social media platforms and what are the complaints about? (iii) Finally, how do veterinarians handle online complaints; do socio‐demographic and practice‐specific factors lead to differences in the way they are handled? Throughout, we highlighted the main similarities and differences among the three countries studied.

## MATERIALS AND METHODS

### Study population and recruitment

In cooperation with small animal veterinary associations in Austria (Vereinigung Österreichischer Kleintiermediziner), Denmark (Danish Veterinary Association) and the UK (British Small Animal Veterinary Association) the link to the online questionnaires was sent to 1195 Austrian, 1287 Danish and 5138 UK small animal veterinarians. For Denmark and Austria, data were collected from 2 March to 9 April 2020. For the UK, the survey was open between 30 March and 7 May 2020. Reminder e‐mails were sent 2 weeks after opening the survey. The study received ethical approval from the Research Ethics Committee of Science and Health at the University of Copenhagen (Reference 504‐0114/19‐5000).

### Study participants

A total of 648 veterinarians completed the questionnaire. Information about socio‐demographic and practice‐specific factors are given for the whole study population and for each sub‐population, in Supporting Information 1.

### Survey development

The questions related to the use of Facebook by veterinary practices were developed based on results of an Austrian focus group study,^20^ a review of empirical studies^8^, ^9^ and publications including anecdotal knowledge about veterinarians’ use of social media.^1^, ^2^, ^4^, ^6^, ^10^, ^13^, ^14^ Participants were informed that completion of the questionnaire was voluntary, that responses were anonymous and that no personal information (e‐mail address, etc.) could be traced back to them.^21^

### Survey design and measurements

The present study used a subset of data from a larger questionnaire survey (see Supporting Information 2). The first subset covered questions on socio‐demographic and practice‐specific factors; the second subset focused to the use of Facebook in veterinary practices and evaluations from clients on webpages and/or social media platforms.

The second subset included the following questions: whether veterinarian's work place had an active Facebook page (response options: ‘yes’, ‘no’ and ‘I don't know’). Five statements were presented to identify attitudes towards the use of Facebook. Response options ranged from 1 ‘strongly disagree’ up to 7 ‘strongly agree’ and 8 ‘I don't know’. Veterinarians were also asked how often they found negative comments from clients about their veterinary service on webpages and/or social media platforms. Response options ranged from ‘never’ to ‘more than 10 times per month’, as well as ‘I don't know’ and ‘prefer not to say’. To investigate the nature of the negative comments we formulated seven types of complaints and asked respondents to tick all that were relevant. The final question explored how veterinarians handled complaints, giving five options and respondents could tick all that applied. The answer options ‘I am not in charge of dealing with negative feedback’ and ‘Other’ were additionally provided.

### Data analysis

The software Alchemer®, IBM SPSS Statistics for Macintosh, Version 26.0,^22^ was used for all analyses. Univariate descriptive statistics were presented in tables, figures or text. For bivariate analysis, chi‐squared tests or Kruskal–Wallis *H* tests were conducted to test whether the frequency distribution differed between the Austrian, Danish and UK sub‐populations (Tables 1, 2, 3, 4, 5). Bonferroni correction was applied for all multiple comparisons. The significance level was *p* < 0.05.

For each country, five ordinal regression analyses were conducted to identify whether socio‐demographic and practice‐specific factors had an impact on veterinarians’ attitudes towards Facebook. The five statements listed in Table 2 were inserted as dependent variables. Explanatory variables (all categorical) inserted in the regression analyses were gender (1 = male, 2 = female), business type (1 = independently owned, 2 = corporate owned), employment type (1 = self‐employed, 2 = employed), involvement in daily management (1 = yes, 2 = no) and having an active Facebook page for the practice (1 = yes, 2 = no). Age was inserted as a continuous variable (range: 23–83 years). Respondents that answered ‘Other’ for business and employment type as well as ‘I don't know’ for having a Facebook page were excluded from these analyses.

Further, for each country, we conducted an ordinal regression analysis to examine whether socio‐demographic and practice‐specific factors had an impact on how often veterinarians found complaints from clients on webpages and/or social media platforms. Frequency of finding complaints was inserted as dependent variable (1 = never, 2 = less than once up to two times per month, 3 = 3 up to more than 10 times per month). The same explanatory variables were inserted as for the above‐described ordinal regression analyses.

For each country, four binary regression analyses were run to examine whether veterinarians’ work experience, employment status and business type were associated with the answer option ‘I am not in charge of dealing with negative feedback’ and with three approaches to the handling of online complaints: (a) ‘I ignore the feedback’, (b) ‘If possible, I reply to every piece of negative feedback’ and (c) ‘If the clients come back to me, I discuss the negative feedback with them’. In all cases the dependent variable was binary (1 = the approach to handle complaints is used, 0 = not used). Gender (1 = male, 2 = female), employment status (1 = self‐employed, 2 = employed), business type (1 = independently owned, 2 = corporate owned) were inserted as categorical explanatory variables. Years of work experience was inserted as continuous explanatory variable (range 0.5–48 years of experience). Respondents that answered ‘Other’ for business and employment type were excluded from these analyses.

Since only two Austrian respondents indicated that they worked in a corporate‐owned practice, the business type variable was excluded from all multi‐variables analyses of Austrian veterinarians.

## RESULTS

### The use of Facebook in small animal practices

Significant differences were identified between Austrian, Danish and UK veterinarians in terms of Facebook use by veterinary practices (Table 1). Veterinarians from Denmark (90.6%) and the UK (88.9%) more often indicated that their workplace had an active Facebook page, compared to Austrian colleagues (43.1%).

**TABLE 1:** Prevalence of active Facebook pages for veterinary practices in Austria (AT) , Denmark (DK) and the UK

	All countries *N* = 643 (%)	Austria *n* = 102 (%)	Denmark *n* = 171 (%)	UK *n* = 370 (%)	Test^a^
Yes	528 (82.1)	44 (43.1)	155 (90.6)	329 (88.9)	*χ* ^2^(2) = 150.474, *p* < 0.001 AT versus DK *χ* ^2^(1) = 74.926, *p* < 0.001 AT versus UK *χ* ^2^(1) = 125.203, *p* < 0.001 UK versus DK *χ* ^2^(1) = 0.148, *p* = 0.701
No	101 (15.7)	58 (56.9)	15 (8.8)	28 (7.6)	
I don't know	14 (2.2)	0 (0.0)	1 (0.6)	13 (3.5)	

*Note*: The (1) and (2) after by the chi square symbol is the information about the df (degree of freedom), which indicates the number of variables included.

^a^ Pearson chi‐squared test (answer option ‘I don't know’ was excluded for this analysis).

### Veterinarians’ attitudes towards Facebook

Fewer Austrian veterinarians agreed with the statements that Facebook ‘gives clients an insight into what happens with the patient in the practice/clinic/hospital’ and ‘is a vital way to recruit new clients’ (37.3% and 22.6%, respectively), compared to veterinarians from Denmark (75.5% and 67.2%) and the UK (65.4% and 54.0%) (Table 2). Similarly, fewer Austrian veterinarians agreed with the statements that Facebook ‘is an important way of communicating for veterinarians and their clients’ and ‘is expected by clients’ (26.5% and 31.7%, respectively), compared to Danish (66.9% and 77.8%) and UK colleagues (70.2% and 80.4%). Conversely, more Austrian veterinarians (14.8%) agreed that a Facebook page ‘is of no relevance’ compared to veterinarians from Denmark (1.8%) and the UK (4.1%).

**TABLE 2:** Attitudes towards the use of Facebook by veterinary practices

No.	Having a Facebook page for the practice …	Level of agreement^a^	Austria *n* = 101–102 (%)	Denmark *n* = 169–171 (%)	UK *n* = 367–368 (%)	Test^b^
1	… gives clients an insight into what happens with the patient in the practice/clinic/hospital.	Disagreement	39 (38.2)	16 (9.3)	53 (14.5)	*H*(2) = 56.181, *p* < 0.001 AT versus DK *p* < 0.001^c^ AT versus UK *p* < 0.001^c^ DK versus UK *p* < 0.001^c^
		Neutral	21 (20.6)	18 (10.5)	62 (16.9)	
		Agreement	38 (37.3)	129 (75.5)	240 (65.4)	
		I don't know	4 (3.9)	8 (4.7)	12 (3.3)	
		Mean ± SD	3.99 ± 1.56	5.40 ± 1.35	4.88 ± 1.38	
2	… is of no relevance.	Disagreement	55 (53.9)	147 (87.0)	282 (76.6)	*H*(2) = 34.391, *p* < 0.001 AT versus DK *p* < 0.001^c^ AT versus UK *p* = 0.001^c^ DK versus UK *p* = 0.001^c^
		Neutral	28 (27.5)	15 (8.9)	50 (13.6)	
		Agreement	15 (14.8)	3 (1.8)	28 (7.6)	
		I don't know	4 (3.9)	4 (2.4)	8 (2.2)	
		Mean ± SD	3.19 ± 1.62	2.12 ± 1.09	2.55 ± 1.27	
3	… is a vital way to recruit new clients.	Disagreement	55 (54.0)	16 (9.4)	74 (20.1)	*H*(2) = 72.619, *p* < 0.001 AT versus DK *p* < 0.001^c^ AT versus UK *p* < 0.001^c^ DK versus UK *p* < 0.001^c^
		Neutral	20 (19.6)	34 (19.9)	80 (21.7)	
		Agreement	23 (22.6)	115 (67.2)	199 (54.0)	
		I don't know	4 (3.9)	6 (3.5)	15 (4.1)	
		Mean ± SD	3.35 ± 1.69	5.06 ± 1.29	4.52 ± 1.37	
4	… is expected by clients.	Disagreement	41 (40.6)	7 (4.1)	21 (5.7)	*H*(2) = 83.217, *p* < 0.001 AT versus DK *p* < 0.001^c^ AT versus UK *p* < 0.001^c^ DK versus UK *p* = 0.869^c^
		Neutral	22 (21.8)	21 (12.3)	39 (10.6)	
		Agreement	32 (31.7)	133 (77.8)	297 (80.4)	
		I don't know	6 (5.9)	10 (5.8)	12 (3.3)	
		Mean ± SD	3.79 ± 1.66	5.50 ± 1.18	5.38 ± 1.19	
5	… is an important way of communicating for veterinarians and their clients.	Disagreement	54 (52.9)	20 (11.8)	52 (14.1)	*H*(2) = 75.817, *p* < 0.001 AT versus DK *p* < 0.001^c^ AT versus UK *p* < 0.001^c^ DK versus UK *p* = 1.00^c^
		Neutral	17 (16.7)	32 (18.9)	50 (13.6)	
		Agreement	27 (26.5)	113 (66.9)	158 (70.2)	
		I don't know	4 (3.9)	4 (2.4)	7 (1.9)	
		Mean ± SD	3.42 ± 1.59	5.01 ± 1.36	5.03 ± 1.45	

Abbreviations: AT, Austria; DK, Denmark.

^a^ Disagreement = 1 ‘strongly disagree’, 2 ‘disagree’ and 3 ‘somewhat disagree’; Neutral = 4 ‘neutral (neither agree nor disagree)’; Agreement = 5 ‘somewhat agree’, 6 ‘agree’ and 7 ‘strongly agree’.

^b^ Kruskal–Wallis *H* test (answer option ‘I don't know’ was excluded from these analyses).

^c^ Bonferroni correction was applied for multiple tests.

### What explains veterinarians’ attitudes towards Facebook?

Ordinal regression analyses indicated that in particular veterinarians’ age and having an active Facebook page for the practice had an impact on their attitudes (Supporting Information 1). Veterinarians from all three countries who work in a practice that actively used Facebook were more likely to agree that it ‘is a vital way to recruit new clients’ (*p*_AT _< 0.001; *p*_DK _= 0.003; *p*_UK _= 0.014) and ‘is expected by clients’ (*p*_AT_ < 0.001; *p*_DK_ < 0.001; *p*_UK_ < 0.001). In addition, younger veterinarians from Denmark and the UK were more inclined to agree that Facebook ‘is a vital way to recruit new clients’ compared to older colleagues (*p*_DK_ = 0.040; *p*_UK_ = 0.046). Veterinarians not using Facebook in their practice were more likely to agree that Facebook ‘is of no relevance’ in Austria, Denmark and the UK (*p*_AT_ < 0.001; *p*_DK_ < 0.001; *p*_UK_ = 0.002) as were older veterinarians in the UK (*p* = 0.020). For Denmark, younger (*p* = 0.019) and male veterinarians (*p* = 0.025) as well as veterinarians that worked in a practice with a Facebook page (*p* = 0.012) were more likely to agree that it ‘gives clients insights into what happens with the patient’. That Facebook is an important way to communicate with clients obtained significantly more agreement from veterinarians who worked in a practice with an active Facebook page (*p*_AT_ < 0.001; *p*_DK_ < 0.001; *p*_UK_ = 0.002).

### Frequency of complaints on the internet and/or social media platforms

We identified differences among Austrian, Danish and UK veterinarians in the reported frequency of online complaints (Table 3). A greater number of Danish and Austrian veterinarians (31.8% and 36.0%, respectively) indicated that they *never* found complaints on the internet, compared to their colleagues from the UK (11.7%). Sixteen percent of UK veterinarians stated that they found complaints one to two times per month compared to Austrian (5.0%) and Danish respondents (3.5%). However, more Danish veterinarians (10.6%) chose the option ‘5–10 times per month’ compared to Austrian (1.0%) and UK (0.8%) veterinarians.

**TABLE 3:** Prevalence of complaints on the internet and/or social media platforms from clients about the veterinarian's service in Austria (AT), Denmark (DK) and the UK

	All countries *N* = 639 (%)	Austria *n* = 100 (%)	Denmark *n* = 170 (%)	UK *n* = 369 (%)	Test^a^
Never	133 (20.8)	36 (36.0)	54 (31.8)	43 (11.7)	*χ* ^2^(2) = 45.150, *p* < 0.001 AT versus DK *χ* ^2^(1) = 0.508, *p* = 0.476 AT versus UK *χ* ^2^(1) = 33.296, *p* < 0.001^b^ DK versus UK *χ* ^2^(1) = 31.898, *p* < 0.001^b^
Less than once per month	308 (48.2)	48 (48.0)	91 (53.5)	169 (45.8)	*χ* ^2^(2) = 2.787, *p* = 0.248
1–2 times per month	70 (11.0)	5 (5.0)	6 (3.5)	59 (16.0)	*χ* ^2^(2) = 22.832, *p* < 0.001 AT versus DK *χ* ^2^(1) = 0.348, *p* = 0.555 AT versus UK *χ* ^2^(1) = 8.036, *p* < 0.015^b^ DK versus UK *χ* ^2^(1) = 17.037, *p* < 0.001^b^
3–4 times per month	17 (2.7)	1 (1.0)	1 (0.6)	15 (4.1)	*χ* ^2^(2) = 6.695, *p* = 0.070^b^
5–10 times per month	22 (3.4)	1 (1.0)	18 (10.6)	3 (0.8)	*χ* ^2^(2) = 35.581, *p* < 0.001 AT versus DK *χ* ^2^(1) = 8.848, *p* = 0.009^b^ AT versus UK *χ* ^2^(1) = 0.033, *p* = 0.857 DK versus UK *χ* ^2^(1) = 29.701, *p* < 0.001^b^
More than 10 times per month	4 (0.6)	0 (0.0)	0 (0.0)	4 (1.1)	*χ* ^2^(2) = 1.857, *p* = 0.173
I don't know	84 (13.1)	9 (9.0)	0 (0.0)	75 (20.3)	*χ* ^2^(2) = 40.138, *p* < 0.001 AT versus DK *χ* ^2^(1) = 15.828, *p* < 0.001^b^ AT versus UK *χ* ^2^(1) = 06.864, *p* = 0.027^b^ DK versus UK *χ* ^2^(1) = 40.138, *p* < 0.001^b^
Prefer not to say	1 (0.2)	0 (0.0)	0 (0.0)	1 (0.3)	*χ* ^2^(2) = 0.733, *p* = 0.639

*Note*: The (1) and (2) after by the chi square symbol is the information about the df (degree of freedom), which indicates the number of variables included.

^a^ Pearson chi‐squared test.

^b^ Bonferroni correction was applied for multiple tests.

In general, socio‐demographic and practice‐specific factors did not explain the prevalence of negative comments except for one instance in Austria (*χ* ^2^(5) = 21.268, *p* = 0.001) (Supporting Information 1); veterinarians who work in a practice with a Facebook page more often found negative comments (*p* = 0.001).

### Reasons for complaints from clients

Veterinarians who reported receiving online complaints from clients were subsequently asked what the complaints were about (Table 4). In general, complaints about the cost of treatment had the highest prevalence and complaints about the lack of technical equipment in the practice had the lowest prevalence in all three countries. Further, UK veterinarians reported significantly more often than their Danish colleagues that clients complained that they had to wait too long (*p* = 0.015). Complaints regarding communication were significantly more often reported by Danish than Austrian veterinarians (*p* = 0.009).

**TABLE 4:** Client's complaints about veterinary service on webpages and/or social media platforms for Austria (AT), Denmark (DK) and the UK

No.	Clients …	Austria *n* = 64 (%)	Denmark *n* = 115 (%)	UK *n* = 323 (%)	Test^a^
1	… complaining about the cost of treatment.	41 (64.1)	74 (64.3)	239 (74.0)	*χ* ^2^(2) = 5.266, *p* = 0.072
2	… complaining about communication.	12 (18.8)	47 (40.9)	105 (32.5)	*χ* ^2^(2) = 9.157, *p* = 0.010 AT versus DK *χ* ^2^(1) = 9.104, *p* = 0.009^b^ AT versus UK *χ* ^2^(1) = 4.793, *p* = 0.087^b^ UK versus DK *χ* ^2^(1) = 2.617, *p* = 0.106
3	… having to wait too long.	8 (12.5)	14 (12.2)	80 (24.8)	*χ* ^2^(2) = 11.078, *p* = 0.004 AT versus DK *χ* ^2^(1) = 0.004, *p* = 0.949 AT versus UK *χ* ^2^(1) = 4.576, *p* = 0.096^b^ UK versus DK *χ* ^2^(1) = 7.980, *p* = 0.015^b^
4	… disagreeing with my medical advice.	16 (25.0)	24 (20.9)	60 (18.6)	*χ* ^2^(2) = 1.466, *p* = 0.480
5	… complaining about specific staff member(s).	13 (20.3)	23 (20.0)	63 (19.5)	*χ* ^2^(2) = 0.029, *p* = 0.985
6	…. complaining about the handling of the patient.	6 (9.4)	25 (21.7)	47 (14.6)	*χ* ^2^(2) = 5.462, *p* = 0.065
7	… complaining about the lack of technical equipment in the practice/clinic/hospital.	0	1 (0.9)	2 (0.6)	*χ* ^2^(2) = 530, *p* = 0.767
8	I don't know.	12 (18.8)	28 (24.3)	56 (17.3)	*χ* ^2^(2) = 1.701, *p* = 0.259
9	Other	9 (14.1)	5 (4.3)	21 (6.5)	*χ* ^2^(2) = 6.292, *p* = 0.043 AT versus DK *χ* ^2^(1) = 5.382, *p* = 0.060^b^ AT versus UK *χ* ^2^(1) = 4.270, *p* = 0.117^b^ UK versus DK *χ* ^2^(1) = 0.705, *p* = 0.401

*Note*: The (1) and (2) after by the chi square symbol is the information about the df (degree of freedom), which indicates the number of variables included.

^a^ Pearson chi‐squared test.

^b^ Bonferroni correction was applied for multiple tests.

### Handling complaints from clients

All respondents who indicated they had received complaints were asked to indicate how they handled complaints from a list of several reply options. UK veterinarians significantly more often indicated that they were not in charge of handling negative feedback (68.6%), compared to their Austrian (23.4%) and Danish (25.4%) colleagues (*χ* ^2^(2) = 88.811, *p* < 0.001).

Table 5 lists the prevalence of responses for six other reply options that provide an insight into how veterinarians that are in charge of feedback handled complaints. The results indicate that veterinarians from all three countries often replied to every piece of negative comment (AT: 36.5%, DK: 52.7%, UK: 42.9%) and often discussed the negative feedback directly with clients who came back (AT: 38.5%, DK: 55.9%, UK: 42.9%). Veterinarians less frequently ignored the feedback (AT: 26.9%, DK: 16.1%, UK: 18.3%) or tried to delete it (AT: 9.6%, DK: 12.9%, UK: 4.8%). No significant differences could be identified among the three countries in relation to the way negative feedback was handled.

**TABLE 5:** Veterinarians handling of online complaints from clients about veterinary services

No.	Veterinarians’ handling of complaints from clients	Austria *n* = 52 (%)	Denmark *n* = 93 (%)	UK *n* = 126 (%)	Test^a^
1	I ignore the feedback.	14 (26.9)	15 (16.1)	23 (18.3)	*χ* ^2^(2) = 2.639, *p* = 0.267
2	If possible I delete the feedback or try to get it deleted.	5 (9.6)	12 (12.9)	6 (4.8)	*χ* ^2^(2) = 4.672, *p* = 0.097
3	If possible I reply to any feedback that I feel is unfair.	9 (17.3)	24 (25.8)	25 (19.8)	*χ* ^2^(2) = 1.773, *p* = 0.412
4	If possible I reply to every piece of negative feedback.	19 (36.5)	49 (52.7)	54 (42.9)	*χ* ^2^(2) = 3.959, *p* = 0.138
5	If the clients come back to me, I discuss the negative feedback with them.	20 (38.5)	52 (55.9)	54 (42.9)	*χ* ^2^(2) = 5.336, *p* = 0.069
6	Other	5 (9.6)	8 (8.6)	13 (10.3)	*χ* ^2^(2) = 0.182, *p* = 0.913

*Note*: Three Austrian, eight Danish and 25 UK veterinarians chose besides the option of ‘I am not in charge’ at least one further option presented in the table.

The (1) and (2) after by the chi square symbol is the information about the df (degree of freedom), which indicates the number of variables included.

^a^ Pearson chi‐squared tests.

### What impacts why veterinarians are not in charge of dealing with complaints?

The binary regression models were significant for Austria (*χ* ^2^(3) = 25.353, *p* < 0.001), Denmark (*χ* ^2^(4) = 25.299, *p* < 0.001) and the UK (*χ* ^2^(4) = 49.051, *p* < 0.001) (Supporting Information 1). Results indicated that employed veterinarians in all three countries were more likely to reply that they were not in charge of dealing with negative feedback, compared to self‐employed veterinarians (*p*_AT _= 0.009; *p*_DK _= 0.007; *p*_UK _< 0.001). Further, in the UK, more experienced veterinarians were more likely to reply that they were not in charge (*p* = 0.006).

### What impacts how complaints are handled?

Two binary regression models were significant; one for Austria (*χ* ^2^(3) = 15.745, *p* = 0.001) and one for UK (*χ* ^2^(4) = 10.962, *p* = 0.027) (Supporting Information 1). For Austria, results indicated that female (*p* = 0.008) and more experienced (*p* = 0.004) veterinarians were more likely to reply to every piece of negative feedback. In the UK, veterinarians who worked in corporate‐owned practices were more likely to ignore the negative feedback compared to their colleagues working in independently‐owned practices and clinics (*p* = 0.004).

## DISCUSSION

With uptake of just over 40%, Austrian practices make considerably less use of Facebook compared to those in Denmark and the UK, where uptake is around 90%. A likely explanation for this relates to differences in the general use of internet services in the three countries. According to the Digital Economy and Society Index,^23^ that assesses the progress of digitalisation of European countries, Denmark and UK are ranked fourth and fifth, respectively, whereas Austria is ranked 19th in regard to the use of internet services.

Not surprisingly, the active use of Facebook affected veterinarians attitudes towards it. Thus, we found that veterinarians from all three countries who do not use Facebook for their veterinary practice were more likely to agree that the use of the platform is of no relevance. Conversely, veterinarians working in practices with a Facebook page were more likely to agree that it was expected by clients or is a vital way to recruit new clients. These findings confirm our assumption that the use of Facebook in veterinary practice and experience gained with it, leads to a stronger belief in the benefits it offers. Relatedly, another study^4^ stated that clients increasingly expect to communicate with their veterinarians via social media platforms and concluded that it is harder for veterinarians who do not make use of such platforms to compete in the veterinary market.

As expected, we found that younger veterinarians in particular, from both the UK and Denmark, were more likely to agree that Facebook was a vital way of recruiting new clients. Younger Danish respondents agreed more strongly than their older colleagues that Facebook provided the opportunity to give clients insights into what happens with the patients in their practices. This generational difference probably reflects the fact that younger people have grown up with digital communications—are so‐called ‘digital natives’—and are familiar with online platforms.^24^ It can be expected that in the near future veterinary practices will be run by digital natives and the distinction in attitudes between younger and older veterinarians is likely to fade out.^24^ In the meantime, it may be a good idea in some cases to give younger colleagues the responsibility for maintaining Facebook pages for veterinary practices.^6^ However, although they may not need training in the mechanics of using social media, they may benefit from being trained in how to use it in a professional context.

Publications on the use of social media by veterinary practices have highlighted the increasing problem of complaints from clients that can be easily distributed online.^2^, ^9^, ^10^ In general, our results showed that UK veterinarians were on average more often confronted with online complaints compared to their Danish and Austrian colleagues. Importantly, although over half of Danish respondents find complaints less than once per month, a subgroup experienced multiple negative comments (5–10 times per month). The contrast between Austria and the two other countries can be explained by the fact that veterinarians and their clients less often engage via Facebook in Austria. The difference between Denmark and the UK in relation to the subgroup of veterinarians finding multiple negative comments came as a surprise because we found a similar share of clinics with Facebook pages in the two countries. A possible explanation for this might be that this subgroup of Danish veterinarians may be more proactive in checking for complaints from clients on the internet, or that clients in Denmark are simply more active in making use of online feedback compared to UK clients. Further insights could be gained through studies with owners.

In all three countries, the most frequent complaints received by veterinarians were about treatment costs. This is not particularly surprising, since complaints about costs of veterinary care,^25^ discussions with clients about costs^26^, ^27^ and the problem of financially limited clients^20^, ^28^, ^29^, ^30^ are among the most pressing concerns in small animal practice. However, as the number of insured animals is relatively high in the UK and Denmark, compared to Austria, potentially reducing the problem of client's financial limitations, we would have expected veterinarians in the UK and Denmark to receive fewer complaints about costs compared to Austrian veterinarians.^31^, ^32^, ^33^

Although the frequency of online complaints varied depending on the nature of the complaint, there is no doubt that clients’ ability to post complaints online may have an impact on practicing veterinarians. In one study on the effect of client complaints on 92 small animal veterinary internists,^25^ 13% of complaints were received online; these in particular caused greater stress and anxiety for veterinarians. Since the use of social media and internet resources will increase in the near future, further research is needed to not only investigate reasons for (online) complaints, but also to examine possible negative effects on veterinarians.

Significantly more of the UK veterinarians in this survey were not in charge of dealing with negative feedback compared to their Austrian and Danish colleagues. This may seem surprising, since almost as many UK veterinarians have a Facebook page for their practice/clinic as do their Danish colleagues and many more compared to Austrian veterinarians. This difference is partially explained by the fact that more of the surveyed UK veterinarians are employed (73.0%) compared to Austria (20.0%) and Denmark (61.0%) and employed veterinarians were less likely to be in charge of dealing with negative comments, while veterinarians that were self‐employed were more likely to deal with them. However, this does not explain all the difference, since over 40% of self‐employed veterinarians in the UK are not in charge of dealing with complaints, compared to 9% of self‐employed veterinarians in Austria and 2% in Denmark. It may be the case in the UK, for example, that dealing with complaints is the responsibility of a non‐veterinarian within the practice, for example, a practice manager.

Several studies have addressed the ways in which veterinarians can manage client complaints.^2^, ^6^, ^34^ For instance, one study^2^ suggested that veterinarians should respond to complaints to show that they care about negative feedback and should encourage other clients to write positive, defending commentaries. Our study results show that many respondents from all three countries took such an active approach, by replying to every piece of negative feedback or discussing the complaints in person if the clients came back.

Furthermore, we found that Austrian veterinarians with more work experience were more likely to reply to every piece of negative feedback. It may be that veterinarians who have worked longer in practice have already built up a loyal clientele and a good reputation that they do not want damaged by online complaints; hence they put more effort in replying to every piece of negative feedback. In addition, our results indicate that UK veterinarians who worked in independently‐owned practices were less likely to ignore the negative feedback compared to their colleagues in corporate‐owned practices. A plausible explanation for this might be that loss of clients has a more direct and potentially devastating effect on veterinarians in independently‐owned practices, compared to those with a company providing financial support in the background. However, it may also be the case that veterinarians who work in corporate‐owned practices receive support from the corporation^35^ to protect the reputation not only of the veterinarian but also of the company, with complaints being addressed centrally rather than the responsibility falling on the individual veterinarian.

Although this study involved three different countries and opens up a novel research field, the study is subject to a number of limitations. The number of participants of the three sub‐populations was disproportionate; in particular the small sample size in Austria could have had an effect on the identification of significant differences in our regression analyses.

Giving the respondents the option to decline to indicate the number of complaints they received each month may introduce bias, as this option is more likely to be chosen by veterinarians who receive a larger number of complaints, or, alternatively, are unaware of the numbers. In addition, our study focused on attitudes to the use of Facebook, but the popularity of different platforms is constantly changing. One study found that Twitter users post many comments that reference veterinarians^36^ for example; we therefore recommend future studies incorporate a wider range of social media platforms. In addition, complaints about communication can have different aspects, such as too little communication, miscommunication or style of communication. Therefore, we recommend exploring complaints in more detail in future work. Further research should also explore potential benefits that may arise from the use of social media such as the recruitment of new clients, in relation to the time invested in maintaining social media platforms. Such information can provide valuable information for veterinary professionals in regard to the successful use and value of social media platforms for their business.

## CONFLICT OF INTEREST

The authors declare they have no conflicts of interest.

## ETHICAL APPROVAL

The study received ethical approval from the Research Ethics Committee of Science and Health at the University of Copenhagen (Reference 504‐0114/19‐5000).

## FUNDING INFORMATION

Austrian Science Fund (FWF), grant number P 29974‐G24.

## Supporting information

Supporting Information 1

Supporting Information 2

## Data Availability

All data are fully available without restrictions.
