# Supplementary material for: Digital opportunities to connect and complain – the use of Facebook in small animal practice
Source: Vet Rec Open. 2022 Feb 28;9(1):e29. doi: 10.1002/vro2.29 (PMC8885750; doi:10.1002/vro2.29)
Supplement: Supplementary file 1 — Supporting Information 1 [file VRO2-9-e29-s002.pdf]

Supporting Information 1

Socio-demographic and practice-specific factors for the whole study population, and for each sub-population from Austria, Denmark and the UK

	All countries (N=615-648)	Austria (n=95-102)	Denmark (n=161-172)	UK (n=359-374)	Tests
**GENDER**					$\chi^2(2)=2.466$, p=0.291
Male	643 (28.1)	25 (24.8)	43 (25.0)	113 (30.5)	
Female	462 (71.9)	76 (75.2)	129 (75.0)	257 (69.5)	
**AGE (in years)**					H(2)=5.316, p=0.070
23-30	102 (16.6)	5 (5.3)	20 (12.4)	77 (21.4)	
31-50	292 (47.5)	55 (57.9)	80 (49.7)	157 (43.7)	
>50	221 (35.9)	35 (36.8)	61 (37.9)	125 (34.8)	
**WORK EXPERIENCE (in years)**					H(2)=0.967, p=0.617
0.5-5	137 (21.3)	11 (11.0)	39 (22.9)	87 (23.3)	
6-10	69 (10.7)	14 (14.0)	19 (11.2)	36 (9.7)	
11-20	135 (21.0)	29 (29.0)	32 (18.8)	74 (19.8)	
≥21-60	302 (47.0)	46 (46.0)	80 (47.1)	176 (47.2)	
**BUSINESS TYPE**					$\chi^2(2)=131.166$, p<0.001, $\phi=.471$<br>AT vs. DK: $\chi^2(1)=14.430$, p<0.001[a], $\phi=.236$<br>AT vs. UK: $\chi^2(1)=91.324$, p<0.001[a], $\phi=.459$<br>DK vs. UK: $\chi^2(1)=65.459$, p<0.001[a], $\phi=.364$
Independently owned	374 (57.7)	97 (95.1)	131 (76.2)	146 (39.0)	
Corporate owned	218 (33.6)	2 (2.0)	28 (16.3)	188 (50.3)	
Other[b] (incl. university and shelter)	56 (8.6)	3 (2.9)	13 (7.6)	40 (10.7)	
**EMPLOYMENT STATUS**					$\chi^2(2)= 107.985$, p<0.001, $\phi=.415$<br>AT vs. DK: $\chi^2(1)=46.230$, p<0.001[a], $\phi=.415$<br>AT vs. UK: $\chi^2(1)=108.329$, p<0.001[a], $\phi=.486$<br>DK vs. UK: $\chi^2(1)=10.577$, p=0.003[a], $\phi=.142$
Self-employed	229 (35.4)	81 (79.4)	63 (36.6)	85 (22.8)	
Employed	397 (61.4)	20 (19.6)	105 (61.0)	272 (72.9)	
Other[b] (retired and unspecified)	21 (3.2)	1 (1.0)	4 (2.3)	16 (4.3)	
**INVOLVEMENT DAILY MANAGEMENT**					χ^2 (2)= 38.877, p<0.001, ϕ= 0.245<br>AT vs. DK: $\chi^2(1)=38.413$, p<0.001[a], $\phi=.374$<br>AT vs. UK: $\chi^2(1)=30.207$, p<0.001[a], $\phi=.252$<br>DK vs. UK: $\chi^2(1)=2.769$, p=0.096
Yes	422 (65.2)	93 (91.2)	95 (55.2)	234 (62.7)	
No	225 (34.8)	9 (8.8)	77 (44.8)	139 (37.3)	

Counts (percentage)

[a] Bonferroni correction was applied for multiple comparison between three countries and significant variables

[b] Answer option "Other" were excluded from bivariate statistics

Ordinal regression analyses of socio-demographic and practice-specific factors on attitudes towards Facebook for small animal practices

**Model 1 AUSTRIA: Having a Facebook page for the practice/clinic/hospital gives clients an insight into what happens with the patient in the practice/clinic/hospital.** ($\chi^2(5)=3.995$, P=0.550)					
			Hypothesis Test		
	B	Std. Error	Wald Chi-Square	df	Sig.
Gender *(ref. cat.: female)*	-0.031	0.4802	0.004	1	0.949
Involvement in daily management *(ref. cat.: no)*	0.365	0.7930	0.212	1	0.645
Employment type *(ref. cat.: employed)*	-0.542	0.6879	0.620	1	0.431
Active Facebook page for practice/clinic/hospital *(ref.cat.: no)*	0.659	0.4047	2.654	1	0.103
age	0.012	0.0282	0.194	1	0.660
**Model 1 DENMARK: Having a Facebook page for the practice/clinic/hospital gives clients an insight into what happens with the patient in the practice/clinic/hospital.** ($\chi^2(6)=14.673$, P=0.023)					
Gender *(ref. cat.: female)*	0.889	0.3970	5.010	1	0.025
Involvement in daily management *(ref. cat.: no)*	-0.055	0.4364	0.016	1	0.901
Business type *(ref. cat.: corporate-owned)*	-0.211	0.4500	0.221	1	0.639
Employment type *(ref. cat.: employed)*	0.094	0.4697	0.040	1	0.842
Active Facebook page for practice/clinic/hospital *(ref.cat.: no)*	1.555	0.6153	6.384	1	0.012
age	-0.038	0.0161	5.464	1	0.019
**Model 1 UK: Having a Facebook page for the practice/clinic/hospital gives clients an insight into what happens with the patient in the practice/clinic/hospital.** ($\chi^2(6)=6.014$, P=0.422)					
Gender *(ref. cat.: female)*	0.180	0.2394	0.567	1	0.452
Involvement in daily management *(ref. cat.: no)*	-0.127	0.2425	0.274	1	0.601
Business type *(ref. cat.: corporate-owned)*	0.118	0.2272	0.268	1	0.605

Employment type *(ref. cat.: employed)*	-0.408	0.3056	1.780	1	0.182
Active Facebook page for practice/clinic/hospital *(ref.cat.: no)*	0.767	0.4648	2.724	1	0.99
age	-0.004	0.0094	0.199	1	0.656
**Model 2 AUSTRIA: Having a Facebook page for the practice/clinic/hospital is of no relevance.** (χ^2(5)=26.836, P<0.001)					
Gender *(ref. cat.: female)*	0.322	0.4760	0.457	1	0.499
Involvement in daily management *(ref. cat.: no)*	0.576	0.7307	0.621	1	0.431
Employment type *(ref. cat.: employed)*	-0.344	0.7056	0.237	1	0.626
Active Facebook page for practice/clinic/hospital *(ref.cat.: no)*	-1.909	0.4514	17.883	1	0.000
age	0.043	0.307	2.006	1	0.157
**Model 2 DENMARK: Having a Facebook page for the practice/clinic/hospital is of no relevance.** (χ^2(6)=21.917, P=0.001)					
Gender *(ref. cat.: female)*	-0.037	0.4039	0.008	1	0.927
Involvement in daily management *(ref. cat.: no)*	-0.427	0.4642	0.847	1	0.357
Business type *(ref. cat.: corporate-owned)*	0.214	0.4703	0.206	1	0.650
Employment type *(ref. cat.: employed)*	0.049	0.4965	0.010	1	0.921
Active Facebook page for practice/clinic/hospital *(ref.cat.: no)*	-2.509	0.6152	16.627	1	0.000
age	0.030	0.0165	3.224	1	0.073
**Model 2 UK: Having a Facebook page for the practice/clinic/hospital is of no relevance.** (χ^2(6)=17.074, P=0.009)					
Gender *(ref. cat.: female)*	0.089	0.2458	0.131	1	0.717
Involvement in daily management *(ref. cat.: no)*	0.447	0.2437	3.358	1	0.067
Business type *(ref. cat.: corporate-owned)*	-0.012	0.2276	0.003	1	0.957

Employment type *(ref. cat.: employed)*	0.079	0.2981	0.070	1	0.791
Active Facebook page for practice/clinic/hospital *(ref.cat.: no)*	-1.467	0.4669	9.880	1	0.002
age	0.022	0.0096	5.442	1	0.020
**Model 3 AUSTRIA: Having a Facebook page for the practice/clinic/hospital is a vital way to recruit new clients.** ($\chi^2(5)$=22.856, P<0.001)					
Gender *(ref. cat.: female)*	-0.326	0.4800	0.462	1	0.497
Involvement in daily management *(ref. cat.: no)*	-0.401	0.8265	0.235	1	0.628
Employment type *(ref. cat.: employed)*	-0.292	0.7003	0.174	1	0.676
Active Facebook page for practice/clinic/hospital *(ref.cat.: no)*	1.678	0.4291	15.283	1	0.000
age	-0.024	0.0297	0.659	1	0.417
**Model 3 DENMARK: Having a Facebook page for the practice/clinic/hospital is a vital way to recruit new clients.** ($\chi^2(6)$=13.502, P=0.036)					
Gender *(ref. cat.: female)*	0.432	0.4120	1.101	1	0.294
Involvement in daily management *(ref. cat.: no)*	-0.089	0.4594	0.038	1	0.846
Business type *(ref. cat.: corporate-owned)*	0.114	0.4645	0.060	1	0.807
Employment type *(ref. cat.: employed)*	0.395	0.4840	0.666	1	0.414
Active Facebook page for practice/clinic/hospital *(ref.cat.: no)*	1.672	0.5603	8.909	1	0.003
age	-0.033	0.0161	4.219	1	0.040
**Model 3 UK: Having a Facebook page for the practice/clinic/hospital is a vital way to recruit new clients.** ($\chi^2(6)$=15.479, P=0.017)					
Gender *(ref. cat.: female)*	0.130	0.2510	0.267	1	0.605
Involvement in daily management *(ref. cat.: no)*	0.174	0.2443	0.506	1	0.477

Business type *(ref. cat.: corporate-owned)*	-0.183	0.2273	0.647	1	0.421
Employment type *(ref. cat.: employed)*	0.058	0.3064	0.036	1	0.849
Active Facebook page for practice/clinic/hospital *(ref.cat.: no)*	1.109	0.4459	6.085	1	0.014
age	-0.019	0.0095	3.983	1	0.046
**Model 4 AUSTRIA: Having a Facebook page for the practice/clinic/hospital is expected by clients.** ($\chi^2(5)=45.426$, P<0.001)					
Gender *(ref. cat.: female)*	0.464	0.4854	0.915	1	0.339
Involvement in daily management *(ref. cat.: no)*	-0.771	0.8188	0.887	1	0.346
Employment type *(ref. cat.: employed)*	-0.752	0.6911	1.185	1	0.276
Active Facebook page for practice/clinic/hospital *(ref.cat.: no)*	2.411	0.4833	24.882	1	0.000
age	-0.038	0.0287	1.720	1	0.190
**Model 4 DENMARK: Having a Facebook page for the practice/clinic/hospital is expected by clients.** ($\chi^2(6)=21.525$, P=0.001)					
Gender *(ref. cat.: female)*	0.011	0.4375	0.001	1	0.981
Involvement in daily management *(ref. cat.: no)*	0.046	0.4673	0.010	1	0.921
Business type *(ref. cat.: corporate-owned)*	0.443	0.4743	0.874	1	0.350
Employment type *(ref. cat.: employed)*	0.469	0.4984	0.886	1	0.347
Active Facebook page for practice/clinic/hospital *(ref.cat.: no)*	2.567	0.6675	14.782	1	0.000
age	0.011	0.0158	0.454	1	0.501
**Model 4 UK: Having a Facebook page for the practice/clinic/hospital is expected by clients.** ($\chi^2(6)=20.647$, P=0.002)					
Gender *(ref. cat.: female)*	0.225	0.2496	0.813	1	0.367

Involvement in daily management *(ref. cat.: no)*	-0.146	0.2446	0.355	1	0.551
Business type *(ref. cat.: corporate-owned)*	-0.097	0.2284	0.179	1	0.672
Employment type *(ref. cat.: employed)*	-0.192	0.3004	0.409	1	0.522
Active Facebook page for practice/clinic/hospital *(ref.cat.: no)*	1.987	0.4828	16.933	1	0.000
age	-0.007	0.0096	0.483	1	0.487
**Model 5 AUSTRIA: Having a Facebook page for the practice/clinic/hospital is an important way of communicating for veterinarians and their clients.** ($\chi^2(5)$=17.971, P=0.003)					
Gender *(ref. cat.: female)*	-0.141	0.4632	0.092	1	0.761
Involvement in daily management *(ref. cat.: no)*	-0.025	0.7651	0.001	1	0.974
Employment type *(ref. cat.: employed)*	-1.068	0.6514	2.687	1	0.101
Active Facebook page for practice/clinic/hospital *(ref.cat.: no)*	1.486	0.4220	12.401	1	0.000
age	0.036	0.0275	1.697	1	0.193
**Model 5 DENMARK: Having a Facebook page for the practice/clinic/hospital is an important way of communicating for veterinarians and their clients.** ($\chi^2(6)$=17.249, P=0.008)					
Gender *(ref. cat.: female)*	0.250	0.4257	0.346	1	0.556
Involvement in daily management *(ref. cat.: no)*	-0.102	0.4514	0.051	1	0.821
Business type *(ref. cat.: corporate-owned)*	0.510	0.4510	1.279	1	0.258
Employment type *(ref. cat.: employed)*	0.483	0.4857	0.989	1	0.320
Active Facebook page for practice/clinic/hospital *(ref.cat.: no)*	2.257	0.6122	13.5888	1	0.000
age	-0.010	0.0158	0.379	1	0.538
**Model 5 UK: Having a Facebook page for the practice/clinic/hospital is an important way of communicating for veterinarians and their clients.** ($\chi^2(6)$=11.847, P=0.065)					

Gender *(ref. cat.: female)*	0.247	0.2443	1.024	1	0.311
Involvement in daily management *(ref. cat.: no)*	-0.170	0.2367	0.519	1	0.471
Business type *(ref. cat.: corporate-owned)*	0.098	0.2284	0.183	1	0.669
Employment type *(ref. cat.: employed)*	0.086	0.3023	0.081	1	0.775
Active Facebook page for practice/clinic/hospital *(ref.cat.: no)*	1.588	0.5151	9.506	1	0.002
age	-0.001	0.0093	0.007	1	0.932

Ordinal regression analyses of socio-demographic and practice-specific factors on frequency of online complaints from clients

**AUSTRIA:** ($\chi^2(5)$=21.268, P=0.001)					
			Hypothesis Test		
	B	Std. Error	Wald Chi-Square	df	Sig.
Gender *(ref. cat.: female)*	0.355	0.6552	0.293	1	0.588
Involvement in daily management *(ref. cat.: no)*	-0.011	1.1721	0.000	1	0.992
Employment type *(ref. cat.: employed)*	-0.652	1.0160	0.411	1	0.521
Active Facebook page for practice/clinic/hospital *(ref.cat.: no)*	1.989	0.5853	11.550	1	0.001
age	-0.041	0.0422	0.940	1	0.332
**DENMARK:** ($\chi^2(6)$=4.372, P=0.626)					
Gender *(ref. cat.: female)*	-0.011	0.4400	0.001	1	0.980
Involvement in daily management *(ref. cat.: no)*	0.508	0.4792	1.123	1	0.289
Business type *(ref. cat.: corporate-owned)*	-0.420	0.5115	0.675	1	0.411
Employment type *(ref. cat.: employed)*	-0.506	0.5148	0.966	1	0.326
Active Facebook page for practice/clinic/hospital *(ref.cat.: no)*	-0.211	0.6659	0.100	1	0.752
age	0.018	0.0171	1.091	1	0.296
**Model UK:** ($\chi^2(6)$=9.278, P=0.159)					
Gender *(ref. cat.: female)*	0.541	0.3780	2.051	1	0.152
Involvement in daily management *(ref. cat.: no)*	-0.167	0.3745	0.200	1	0.655
Business type *(ref. cat.: corporate-owned)*	-0.213	0.3487	0.372	1	0.542
Employment type *(ref. cat.: employed)*	-0.488	0.4471	1.191	1	0.275

Active Facebook page for practice/clinic/hospital *(ref.cat.: no)*	0.427	0.6801	0.395	1	0.530
age	-0.029	0.0150	3.616	1	0.655

Binary regression analyses of socio-demographic and practice-specific factors on how veterinarians handle online complaints from clients

**Model 1 AUSTRIA: I am not in charge of dealing with negative feedback.** (χ^2(3)=25.353, P<0.001)					
			Hypothesis Testing		
	B	Std. Error	Wald Chi-Square	df	Sig.
(Intercept)	-1.483	0.6923	4.590	1	0.032
Gender *(ref. cat.: female)*	-0.910	1.2235	0.553	1	0.457
Employment type *(ref. cat.: employed)*	2.322	0.8889	6.824	1	0.009
Work experience	0.099	0.0540	3.349	1	0.067
**Model 1 DENMARK: I am not in charge of dealing with negative feedback.** (χ^2(4)=25.299, P<0.001)					
(Intercept)	-0.222	0.6793	0.107	1	0.744
Gender *(ref. cat.: female)*	-0.264	0.7311	0.131	1	0.718
Employment type *(ref. cat.: employed)*	2.955	1.0932	7.307	1	0.007
Business type *(ref. cat.: corporate-owned)*	0.144	0.6254	0.053	1	0.817
Work experience	0.039	0.0259	2.260	1	0.133
**Model 1 UK: I am not in charge of dealing with negative feedback.** (χ^2(4)=49.051, P<0.001)					
(Intercept)	-2.197	0.3089	50.572	1	<0.001
Gender *(ref. cat.: female)*	0.544	0.3120	3.044	1	0.081
Employment type *(ref. cat.: employed)*	1.312	0.3477	14.244	1	<0.001
Business type *(ref. cat.: corporate-owned)*	0.420	0.2968	2.002	1	0.157
Work experience	0.034	0.0122	7.699	1	0.006
**Model 2 AUSTRIA: I ignore the feedback.** (χ^2(3)=2.006, P=0.571)					
(Intercept)	0.720	0.8787	0.672	1	0.412
Gender *(ref. cat.: female)*	0.388	0.8496	0.209	1	0.648
Employment type *(ref. cat.: employed)*	1.148	0.9619	1.424	1	0.233
Work experience	-0.043	0.0403	1.119	1	0.290

**Model 2 DENMARK: I ignore the feedback.** ($\chi^2(4)$=3.273, P=0.513)					
(Intercept)	2.851	1.2091	5.561	1	0.018
Gender *(ref. cat.: female)*	0.407	0.7495	0.294	1	0.587
Employment type *(ref. cat.: employed))*	-0.546	0.7080	0.595	1	0.441
Business type *(ref. cat.: corporate-owned)*	-0.971	1.1648	0.695	1	0.404
Work experience	-0.010	0.0286	0.113	1	0.737
**Model 2 UK: I ignore the feedback.** ($\chi^2(4)$=10.962, P=0.027)					
(Intercept)	2.132	0.6903	9.539	1	0.002
Gender *(ref. cat.: female)*	-0.205	0.5667	0.131	1	0.718
Employment type *(ref. cat.: employed))*	1.019	0.6367	2.559	1	0.110
Business type *(ref. cat.: corporate-owned)*	-1.794	0.6277	8.170	1	0.004
Work experience	0.011	0.0239	0.226	1	0.634
**Model 3 AUSTRIA: If possible I reply to every piece of negative feedback.** ($\chi^2(3)$=15.745, P=0.001)					
(Intercept)	0.739	1.1616	0.405	1	0.525
Gender *(ref. cat.: female)*	-2.740	1.0406	6.932	1	0.008
Employment type *(ref. cat.: employed)*	-2.499	1.3369	3.493	1	0.062
Work experience	0.152	0.0530	8.181	1	0.004
**Model 3 DENMARK: If possible I reply to every piece of negative feedback.** ($\chi^2(4)$=0.789, P=0.940)					
(Intercept)	-0.587	0.6819	0.742	1	0.398
Gender *(ref. cat.: female)*	0.168	0.5313	0.100	1	0.752
Employment type *(ref. cat.: employed))*	-0.355	0.5300	0.449	1	0.503
Business type *(ref. cat.: corporate-owned)*	0.506	0.6468	0.612	1	0.434
Work experience	0.008	0.0209	0.139	1	0.710

**Model 3 UK: If possible I reply to every piece of negative feedback.** ($\chi^2(4)$=5.016, P=0.286)					
(Intercept)	0.200	0.4570	0.191	1	0.662
Gender *(ref. cat.: female)*	-0.353	0.4443	0.632	1	0.427
Employment type *(ref. cat.: employed))*	-0.850	0.4687	3.291	1	0.070
Business type *(ref. cat.: corporate-owned)*	0.466	0.4421	1.110	1	0.292
Work experience	0.022	0.0184	1.440	1	0.230
**Model 4 AUSTRIA: If the clients come back to me, I discuss the negative feedback with them.** ($\chi^2(3)$=6.441, P=0.092)					
(Intercept)	-1.016	0.8927	1.297	1	0.255
Gender *(ref. cat.: female)*	-1.584	0.8397	3.560	1	0.059
Employment type *(ref. cat.: employed)*	0.680	0.9284	0.537	1	0.464
Work experience	0.072	0.0395	3.290	1	0.070
**Model 4 DENMARK: If the clients come back to me, I discuss the negative feedback with them.** ($\chi^2(4)$=1.204, P=0.877)					
(Intercept)	-0.231	0.6703	0.119	1	0.730
Gender *(ref. cat.: female)*	0.250	0.5298	0.223	1	0.636
Employment type *(ref. cat.: employed))*	-0.018	0.5313	0.001	1	0.973
Business type *(ref. cat.: corporate-owned)*	-0.307	0.6381	0.232	1	0.630
Work experience	0.011	0.0209	0.262	1	0.609
**Model 4 UK: If the clients come back to me, I discuss the negative feedback with them.** ($\chi^2(4)$=2.128, P=0.712)					
(Intercept)	0.181	0.4493	0.162	1	0.688
Gender *(ref. cat.: female)*	0.496	0.4307	1.327	1	0.249
Employment type *(ref. cat.: employed))*	-0.039	0.4493	0.008	1	0.931
Business type *(ref. cat.: corporate-owned)*	0.292	0.4176	0.489	1	0.484
Work experience	-0.014	0.0178	0.649	1	0.420
